# A Loop-Mediated Isothermal Amplification Assay Utilizing Hydroxy Naphthol Blue (LAMP-HNB) for the Detection of *Treponema pallidum* Subspp. *pallidum*

**DOI:** 10.3390/pathogens13110949

**Published:** 2024-10-31

**Authors:** Saranthum Phurijaruyangkun, Pongbun Tangjitrungrot, Pornpun Jaratsing, Suphitcha Augkarawaritsawong, Khurawan Kumkrong, Sawanya Pongparit, Pawita Suwanvattana, Supatra Areekit, Kosum Chansiri, Somchai Santiwatanakul

**Affiliations:** 1 Faculty of Medicine, Srinakharinwirot University, Bangkok 10110, Thailand; saranthum.phurijaruyangkun@g.swu.ac.th; 2 Center of Excellence in Biosensors, Panyananthaphikhu Chonprathan Medical Center, Srinakharinwirot University, Nonthaburi 11120, Thailand; pongbun.pao@gmail.com (P.T.); pornpunj@g.swu.ac.th (P.J.); supatraa@g.swu.ac.th (S.A.); prof.kosum@gmail.com (K.C.); 3 Faculty of Medical Technology, Rangsit University, Pathum Thani 12000, Thailand; suphitcha.a@rsu.ac.th (S.A.); khurawan.k@rsu.ac.th (K.K.); sawanya.p@rsu.ac.th (S.P.); 4 Medical Technology and Reference Laboratory for Infectious Diseases, Bamrasnaradura Infectious Diseases Institute, Nonthaburi 11000, Thailand; pawitasuwan@gmail.com; 5 Innovative Learning Center, Srinakharinwirot University, Bangkok 10110, Thailand

**Keywords:** *Treponema pallidum*, loop-mediated isothermal amplification (LAMP), hydroxy naphthol blue (HNB)

## Abstract

*Treponema pallidum* subspp. *pallidum* is a spirochaete bacterium that causes syphilis, one of the most common sexually transmitted diseases. Syphilis progresses through four distinct stages, each characterized by specific symptoms, namely primary, secondary, latent, and late (tertiary) syphilis. Serology has been considered the primary diagnostic approach. However, it is plagued by problems such as the limited specificity of nontreponemal tests and the inadequate correlation of treponemal tests with disease activity. In this study, we focused on the development of a loop-mediated isothermal amplification assay utilizing hydroxy naphthol blue (LAMP-HNB) for the diagnosis of *T. pallidum* subspp. *pallidum*. Specifically, this study seeks to determine the analytical sensitivity (limit of detection; LOD) and analytical specificity. Four hundred clinical serum samples were analyzed for diagnostic sensitivity, specificity, and predictive value, and each technique’s 95% confidence intervals (95% CI, *p* < 0.05) were evaluated. The limit of detection for polymerase chain reaction with agarose gel electrophoresis (PCR-AGE), the loop-mediated isothermal amplification assay combined with agarose gel electrophoresis (LAMP-AGE), and LAMP-HNB were 116 pg/µL, 11.6 pg/µL, and 11.6 pg/ µL, respectively. Analytical specificity examinations indicated the absence of cross-reactivity with *Leptospira interrogans, Staphylococcus aureus, Enterococcus faecalis, Escherichia coli, Klebsiella pneumoniae, Acinetobacter baumannii, Pseudomonas aeruginosa,* human immunodeficiency virus (HIV), and healthy human serum in PCR-AGE, LAMP-AGE, and LAMP-HNB. The diagnostic sensitivity, diagnostic specificity, positive predictive value (PPV), and negative predictive value (NPV) for PCR-AGE were 100.00 (100.00)%, 94.50 (94.40–94.60)%, 94.79 (94.69–94.88)%, and 100.00 (100.00)%, respectively. While, for LAMP-AGE and LAMP-HNB, they were 100.00 (100.00)%, 91.00 (90.87–91.13)%, 91.74 (91.63–91.86)%, and 100.00 (100.00)%, respectively. The LAMP-HNB test is simple, rapid, highly sensitive, and highly specific, without requiring expensive equipment. In the future, the LAMP-HNB assay may develop into a single-step diagnostic process, enabling the use as point-of-care testing for the diagnosis, prevention, and management of syphilis infection.

## 1. Introduction

Syphilis is re-emerging around the world; it is one of the most widespread sexually transmitted diseases and is caused by *Treponema pallidum* subspp. *pallidum*, a spiral-shaped bacterium [1,2]. The transmission primarily occurs during sexual activity, with the bacterium entering via mucous membranes of the anus, vagina, penis, mouth, or broken skin. *T. pallidum* can disseminate from the site of infection to another tissue using corkscrew-like motility and produces an enzyme that degrades hyaluronic acid and that helps to spread through blood circulation [3]. Syphilis develops in four stages: primary, secondary, latent, and tertiary, each with a distinct set of symptoms. In primary and secondary stages, the disease is contagious and can be sexually transmitted. Congenital syphilis can also occur via the transmission of the disease from mothers to their fetuses [4,5]. Symptoms such as a chancre, a sore on the genitals, typically appears 2 to 12 weeks after exposure; the disease spreads orally, anally, or vaginally, and the symptoms may occur intermittently [6,7]. In the secondary stage of syphilis, most patients develop a skin rash, but the absence of symptoms does not rule out infection. The disease can progress to a latent, asymptomatic stage if infected persons are without treatment [8]. About 20% of patients develop late-stage syphilis, which is associated with severe health problems like dementia, intellectual disability, heart disease, and nerve damage [9,10]. Syphilis is treated with a single dose of 2.4 million units of benzathine penicillin G via intramuscular injection. There is currently no recommendation to treat syphilis in HIV-infected people with more than one injection. Patients allergic to penicillin may be treated with doxycycline 100 mg twice daily for 14 days [11].

*T. pallidum* subspecies *pallidum* cannot be grown using standard media, and the only isolation method is the rabbit infectivity test [12]. Diagnosing syphilis, especially congenital and neurosyphilis, is challenging, with test interpretation depending on the disease stage. Although serological tests have been essential for screening and monitoring syphilis, they have limitations, including poor correlation between test types [13]. Non-treponemal tests such as rapid plasma reagin (RPR) and venereal disease research laboratory (VDRL) are used for screening, while treponemal tests such as fluorescent treponemal antibody absorption (FTA-ABS), *Treponema pallidum* hemagglutination (TPHA), and *Treponema pallidum* particle agglutination assay (TPPA) are used for confirmation. Serological testing may lack sensitivity in early disease stages, so direct detection tests may be more useful for rapid diagnosis and early treatment [14]. Currently, reverse-sequence algorithms are employed, beginning with an automated treponemal test for screening reactive samples, such as an enzyme immunoassay (EIA) or chemiluminescent immunoassay (CIA). A quantitative non-treponemal test such as RPR or VDRL is conducted if the results are positive. In cases where the reverse-sequence algorithm yields discordant results, a second treponemal test (TPHA/TPPA) is performed to determine the results. Additionally, the reverse-sequence algorithms result in a higher rate of false positives (e.g., EIA, RPR, and TPPA reactions) than the traditional algorithm (0.6%) [15].

The nucleic acid amplification tests (NAATs) include polymerase chain reaction (PCR), and loop-mediated isothermal amplification (LAMP) assays, which are used to detect the bacterial nucleotides. NAATs have been used to develop the detection of pathogenic bacteria, which can be used for on-site diagnostics [16]. Since *T. pallidum* cannot be cultivated, molecular assays involving NAATs for the direct detection of *T. pallidum* subspp. *pallidum* DNA has been utilized to enhance diagnostic sensitivity [17]. In recent years, there has been an increase in the development of techniques for diagnosing syphilis using NAATs to improve the accuracy of the tests. The most used NAAT is the amplification of a specific gene by PCR [18]. The commonly used specific gene for diagnosing syphilis by PCR is the *T. pallidum* 47 kDa lipoprotein (*Tpp47*) gene. This gene encodes a membrane protein involved in cell wall synthesis and has been found to have high sensitivity and specificity in diagnosing syphilis [19,20]. In particular, the LAMP technique is a technique that performs within one hour at constant temperature, relying on the action of the *Bst* DNA polymerase, which has strand-shift activity and is also stable at temperatures between 60 and 65 °C. The portable LAMP detection system involves the relatively straightforward incorporation of deoxynucleotide triphosphates (dNTPs) into the DNA strand during the polymerization process, with one of the secondary products released being pyrophosphate [21,22]. The reaction of the LAMP technique can be monitored by observing the turbidity of pyrophosphate formation and color change tests such as SYBR-Green and Calcein and analyzed by gel electrophoresis. The indicator hydroxy naphthol blue (HNB) has been reported as a highly effective nontoxic alternative for visual assessment [23].

**Figure 1:**
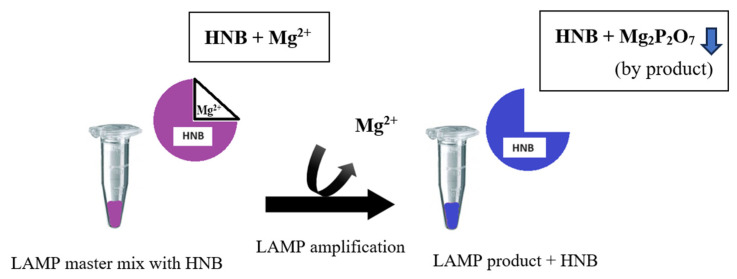
The change in the color of hydroxy naphthol blue (HNB) is attributed to the decrease in magnesium. The concentration of Mg^2+^ in the solution decreases during the LAMP process, the color of the HNB solution changes from purple to blue, which can be observed with the naked eye after amplification.

Loop-mediated isothermal amplification assay utilizing HNB (LAMP-HNB) can be used for visual colorimetric distinction of positive and negative templates. A positive result of the LAMP reaction can produce a large number of pyrophosphate ions, and the compound demonstrates a high reactivity toward Mg^2+^ ions, leading to the precipitation of magnesium pyrophosphate [24,25]. As the concentration of Mg^2+^ in the solution decreases during the LAMP process, the color of the HNB solution changes from purple to blue, which can be observed with the naked eye after amplification. Using colorimetric detection with HNB improves the discrimination between positive and negative samples in the LAMP assay, offering comparable sensitivity to turbidity detection. HNB is a simple, low-cost synthetic dye used in the LAMP method, which is easy to analyze visually (Figure 1) [26,27]. A colored assay has been developed for identifying LAMP reactions using the chromophore HNB, which surpasses the other methods in this field in terms of minimizing contamination risks [28]. In the current study, we aim to develop and evaluate a molecular approach combining LAMP with hydroxy naphthol blue (LAMP-HNB) for the diagnosis of *T. pallidum* subspp. *pallidum*. Specifically, this study seeks to determine diagnostic parameters such as the analytical and diagnostic (clinical) sensitivity and specificity of this assay, targeting the amplification of the gene encoding the 47 kDa membrane immunogen (*Tpp*47), which is highly conserved and specific to *T. pallidum*.

## 2. Materials and Methods

### 2.1. Sample Collection

Four hundred clinical serum samples were systematically collected from patients who were clinically suspected of harboring syphilis at Panyananthaphikkhu Chonprathan Medical Center, Srinakharinwirot University. The diagnostic criteria for syphilis were based on both serological testing and physician assessment (disease or non-disease), with serological testing conducted in accordance with the CDC laboratory guidelines for syphilis; the traditional algorithm for serologic screening begins with a nontreponemal test (RPR), followed by confirmation of RPR reactive samples using a treponemal test (*T. Pallidum* hemagglutination assay; TPHA) [29]. Healthy human serum was obtained from healthy individuals and served as a control group without syphilis. The positive control was the DNA of *T. pallidum*, (ATCC BAA^®^2642SD^TM^). This study was approved by the Institutional Ethics Committee of Srinakharinwirot University (SWUEC/X-099/2565) in April 2022.

### 2.2. DNA Extraction

DNA was extracted from 200 μL of clinical serum samples and healthy human serum using a QIAamp^®^ Blood Mini Kit (Qiagen, Germany), following the manufacturer’s instructions. The samples were eluted in 50 μL AE buffer, and the extracted DNA was at −20 °C until testing.

### 2.3. LAMP Primer Design

The PCR and LAMP primer set used for the amplification of the *Tpp47* gene was designed with the prior analysis of the sequence of *T. pallidum* subspp. *pallidum* (GeneBank accession number:AE000520.1:622266-623570) and used Primer Explorer V5 (https://primerexplorer.jp/e/ (accessed on 10 March 2022)) and Primer3web V4.1.0. software (https://primer3.ut.ee/ (accessed on 10 March 2022). The LAMP primer set consists of two outer primers, F3 and B3, and two inner primers, FIP and BIP, all of which recognize six different sequences on the target DNA. This set comprises primers for which an allowance has been granted for a patent application under submission number 2103001639 (Table 1). The primer/probe analysis of sequences and identification was performed through NCBI nucleotide BLAST with 100% identity to *T. pallidum* subspp. *pallidum* purchased from Pacific Science Co., Ltd., Biobasic, Canada.

### 2.4. The Optimization of PCR Conditions

For target gene amplification by PCR, it is necessary to select the most suitable temperature in the annealing step, which allows the specific primers to bind to the complementary nucleotide sequences and produces the most distinct PCR amplicon bands without any non-specific bands. A temperature gradient of a thermal cycler (C1000 Touch™ Thermal Cycler from Bio-Rad Laboratories Ltd. (Bio-Rad, Hercules, CA, USA)) in the range of 50–60 °C was used to determine the most appropriate annealing temperature for our PCR assay. The PCR amplification was conducted in 25 μL of reaction mixture containing deionized water, 10x PCR buffer (Vivantis, Darul Ehsan, Malaysia) 0.4 μM of F3 and B3 primers, 2.8 mM of MgCl_2_ (Vivantis, Darul Ehsan, Malaysia), 0.2 mM of dNTPs (New England Biolabs, USA), 2 units of Taq DNA polymerase (Vivantis, Darul Ehsan, Malaysia), and 1 μL of DNA template. Milli-Q water was used as a blank negative control. The PCR was performed by using a PCR thermal cycler. The cycles included an initial denaturation step for 5 min at 95 °C, followed by 35 cycles of denaturation at 95 °C for 30 s; the optimal annealing temperature for 30 s; extension at 72 °C for 30 s; and a final extension at 72 °C for 5 min. Subsequently, 192 bp PCR products were analyzed using 2.0% agarose gel electrophoresis (AGE) at 100 volts for 30 min before staining with ViSafe Red Gel Stain (Vivantis, Darul Ehsan, Malaysia); the PCR products of each sample were visualized on a UV transilluminator.

### 2.5. The Optimization of LAMP Reaction Conditions

The LAMP assay’s optimal conditions were determined, including the appropriate amplification temperature, MgSO_4_ concentrations, and the minimum amplification time. The suitable amplification temperature was determined using a gradient thermal cycler (C1000 Touch™ Thermal Cycler from Bio-Rad Laboratories Ltd. (Hercules, CA, USA)) set between 60 and 65 °C. Testing for the appropriate MgSO_4_ concentrations and the cofactor for the *Bst* DNA polymerase enzyme was carried out using varying concentrations of MgSO4 between 3.5 and 6.5 mM. The minimum amplification time required to detect the target gene was tested between 15 and 60 min using a heat block.

For LAMP reactions, a 25 μL reaction volume was used, comprising deionized water, 10x isothermal buffer (New England Biolabs, Ipswich, MA, USA), 2.0 μM each of FIP and BIP, 0.2 μM each of F3 and B3, 1.6 mM of dNTPs (New England Biolabs, USA), 0.5 M betaine (Sigma-Aldrich, USA), optimized MgSO_4_ concentrations (New England Biolabs, USA), 8 U of BstII DNA polymerase (large fragment; New England Biolabs, USA), and 1 μL of DNA. Deionized water was used as a blank negative control to assess and control the risk of sample-handling cross-contamination. The reaction mixture was incubated at the appropriate amplification temperature and reaction times using a heat block. To detect the real-time LAMP product, gel electrophoresis was performed in a 2.0% agarose gel stained with ViSafe Red Gel Stain (Vivantis, Darul Ehsan, Malaysia), electrophoretically run under 100 V for 30 min, and the samples were observed on a UV transilluminator. A ladder-like band pattern on the agarose gel indicated positive amplification.

### 2.6. The Optimization of LAMP-HNB

The optimization of LAMP-HNB was achieved by the addition of an HNB metal indicator (Sigma-Aldrich, St. Louis, MO, USA) to help observe the color change of HNB during the LAMP reaction. The stock HNB was prepared in deionized water to a concentration of 20 mM and diluted with deionized water to obtain a working solution with a concentration ranging from 1 to 20 μM. One mL of each HNB working solution was added into 25 μL of LAMP reaction mixture. The target DNA was quantified by the LAMP assay using the optimal conditions. The LAMP reaction results could be read visually, as positive results would be represented by a change from purple to blue, while negative results would be represented by the color remaining purple.

### 2.7. Analytical Sensitivity and Specificity Tests

To investigate the analytical sensitivity (limit of detection; LOD), the different concentrations of *T. pallidum* ranging from 11.6 ng/µL to 1.16 fg/µL were prepared by 10-fold serial dilutions of the positive DNA of *T. pallidum*, (ATCC BAA^®^2642SD^TM^). These samples were then amplified by both PCR and LAMP assays. The analytical specificity of all assays was assessed through the evaluation of cross-reactivity with *Leptospira interrogans*, *Staphylococcus aureus*, *Enterococcus faecalis*, *Escherichia coli*, *Klebsiella pneumoniae*, *Acinetobacter baumannii*, *Pseudomonas aeruginosa*, HIV, healthy human serum, and deionized water as a negative control.

### 2.8. Diagnostic Accuracy Analysis

The four hundred clinical serum samples included ones positive for syphilis (disease), characterized by RPR reactive, ≥1:16 and TPHA reactive, and negative for syphilis (non-disease), indicated by RPR non-reactive; all samples obtained diagnosis confirmed by a physician (Table 2). The duplicated reactions for clinical serum samples including positive and negative controls were used in all assays. The diagnostic accuracy of tests was calculated using a 2 × 2 cross-tabulation table for the determination of diagnostic sensitivity, diagnostic specificity, positive predictive value (PPV), negative predictive value (NPV), and their 95% confidence intervals (95% CI, *p* < 0.05) by comparing diagnostic test results and true disease status, as shown in Table 3.

## 3. Results

The LAMP assay’s optimal conditions were determined, including the appropriate amplification temperature, MgSO_4_ concentrations, and the minimum amplification time. The suitable amplification temperature was determined using a gradient thermal cycler between 60 and 65 °C. Testing for the appropriate MgSO_4_ concentrations was carried out using varying concentrations of MgSO_4_ between 3.5 and 6.5 mM. The minimum amplification time required to detect the target gene was tested between 15 and 60 min.

### 3.1. The Optimization of the LAMP Assay

**Figure 2:**
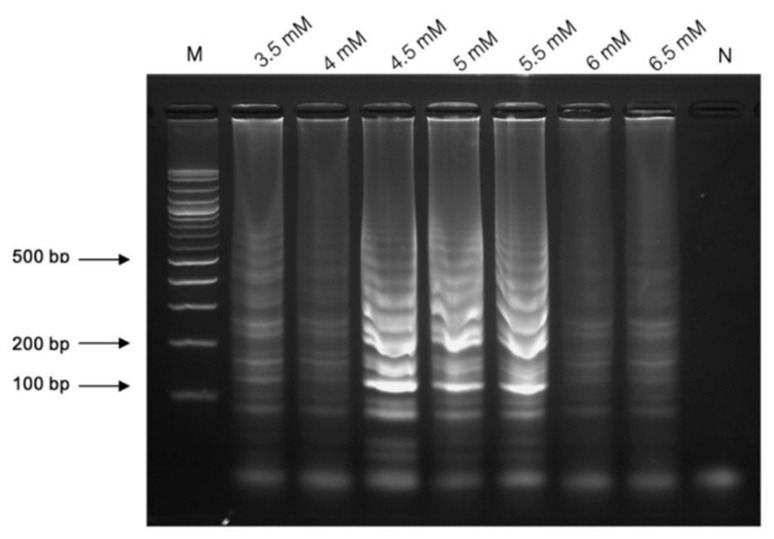
The optimization of the LAMP assay in the range of 4.5–5.5 mM. Lane “M” represents a 100 bp plus DNA ladder marker of Vivantis, Darul Ehsan, Malaysia, and “N” is the negative control.

The optimal concentration of MgSO_4_ for the LAMP was assessed using the concentration between 3.5 and 6.5 mM. The best results were achieved with the MgSO_4_ concentration range of 4.5 to 5.5 mM. In this study, a concentration of 5 mM MgSO_4_ was utilized (Figure 2).

### 3.2. The Optimization of Thermal Conditions

**Figure 3:**
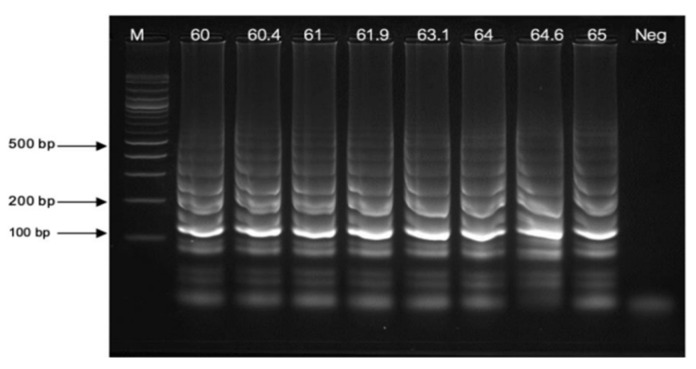
The optimization of thermal conditions ranging between 60 and 65 °C. Lane “M” represents a 100 bp plus DNA ladder marker of Vivantis, Darul Ehsan, Malaysia, and “Neg” is the negative control.

With the temperature conditions ranging between 60 and 65 °C, the test performed at 63 °C yielded the maximum LAMP amplification (Figure 3).

### 3.3. The Optimization of Reaction Durations

**Figure 4:**
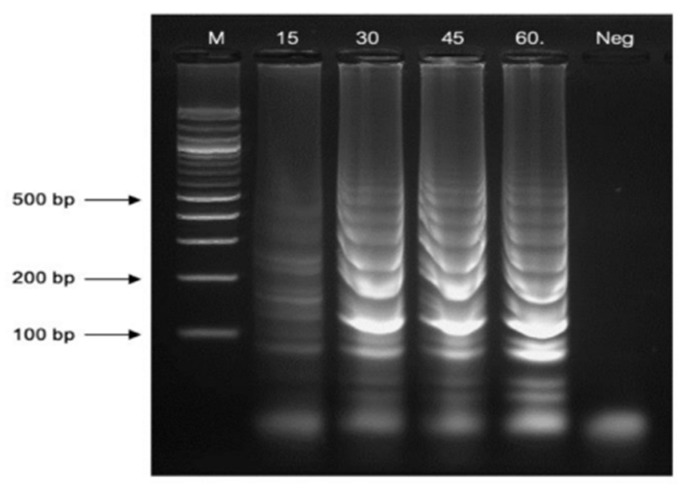
The optimal reaction durations were observed at 45 and 60 min. Lane “M” represents a 100 bp plus DNA ladder marker of Vivantis, Darul Ehsan, Malaysia, and “Neg” is the negative control.

The reaction times for the LAMP assay were evaluated using intervals of 15, 30, 45, and 60 min. The optimal reaction times were observed at 60 min, yielding the highest quantity of LAMP products. (Figure 4).

### 3.4. The Optimization of LAMP-HNB

**Figure 5:**
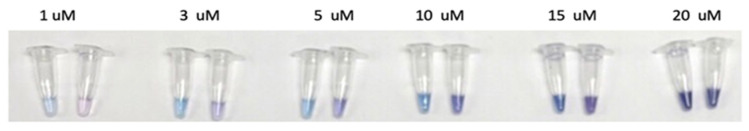
The optimization of LAMP-HNB, ranging from 1 to 20 µM.

The concentrations of HNB ranging from 1 to 20 µM were evaluated. At a concentration of 5 µM, the positive and negative solutions were differentiated by blue and purple color, respectively (Figure 5).

### 3.5. Analytical Sensitivity and Specificity Tests

**Figure 6:**
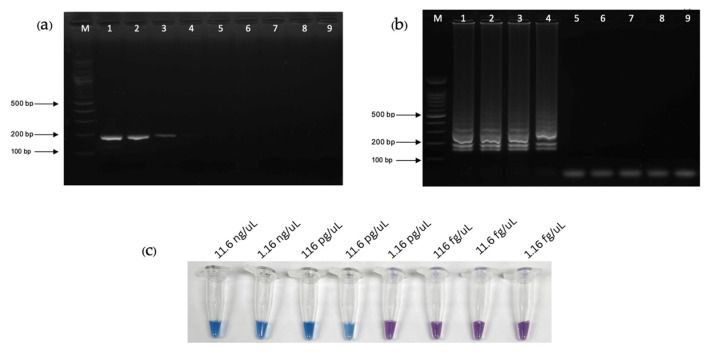
The analytical sensitivity and specificity tests and DNA analysis using 10-fold dilution. In the agarose gel electrophoresis (AGE) results, lanes 1–9: 11.6 ng/µL, 1.16 ng/µL, 116 pg/µL, 11.6 pg/µL, 1.16 pg/µL, 116 fg/µL, 11.6 fg/µL, 1.16 fg/µL, and negative control, respectively. Lane “M” represent a 100 bp plus DNA ladder marker of Vivantis. (**a**) PCR with agarose gel electrophoresis (PCR-AGE). (**b**) LAMP with agarose gel electrophoresis (LAMP-AGE). (**c**) LAMP utilizing hydroxy naphthol blue (LAMP-HNB).

**Figure 7:**
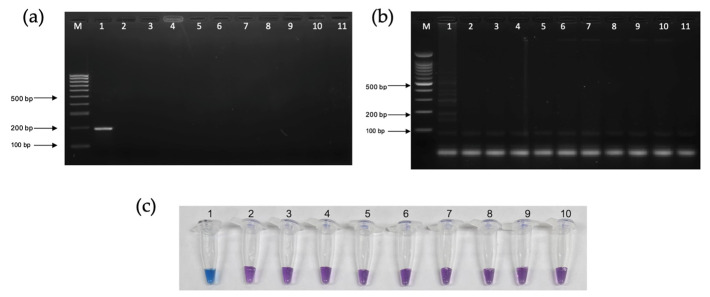
In the agarose gel electrophoresis (AGE) results, lanes 1–11: *T. pallidum* subspp. *pallidum*, *Leptospira interrogans*, *Staphylococcus aureus*, *Enterococcus faecalis*, *Escherichia coli*, *Klebsiella pneumoniae*, *Acinetobacter baumannii*, *Pseudomonas aeruginosa*, Human Immunodeficiency Virus (HIV), healthy human serum, and negative control, respectively. Lane “M” represent a 100 bp plus DNA ladder marker of Vivantis. (**a**) PCR with agarose gel electrophoresis (PCR-AGE). (**b**) LAMP with agarose gel electrophoresis (LAMP-AGE). (**c**) LAMP utilizing hydroxy naphthol blue (LAMP-HNB).

The analytical sensitivity (limit of detection) of the PCR-AGE, LAMP-AGE, and LAMP-HNB assays were 116 pg/µL, 11.6 pg/µL, and 11.6 pg/µL, respectively (Figure 6a–c). Considering specificity, the PCR-AGE, LAMP-AGE, and LAMP-HNB assays exhibited no cross-reactivity with *L. interrogans*, *S. aureus*, *E. faecalis*, *E. coli*, *K. pneumoniae*, *A. baumannii*, *P. aeruginosa*, HIV, and healthy human serum (Figure 7a–c).

### 3.6. Diagnostic Accuracy Analysis

The diagnostic sensitivity, diagnostic specificity, PPV, and NPV of PCR-AGE, LAMP-AGE, and LAMP-HNB, are shown in Table 4, Table 5 and Table 6.

## 4. Discussion

Syphilis is a re-emerging disease, caused by the bacterium *T. pallidum* subspp. *pallidum,* which is transmitted as a sexual disease expectant in multiple sex partners and associated with significant morbidity and mortality. The World Health Organization estimates that in the year 2022, 8 million adults aged 15 to 49 years were infected with syphilis [30]. In Thailand, the studies found that syphilis affected 30,302 Thais, with an incidence rate of 45.2 cases per 100,000 population. The male-to-female ratio was 1:0.8. The highest incidence rate was found in the 15–24-year-old population, at 144.7 cases per 100,000 population. In addition, a 43.6% increase in syphilis cases was reported among temporary or factory workers, and 19.1% of patients were also reported to be co-infected with the human immunodeficiency virus (HIV). It is noteworthy that 3039 cases of syphilis were reported in pregnant women, with an incidence rate of 0.56% and a mean age of 21.2 years [31,32]. The routes of transmission include direct sexual contact with infectious lesions, blood transfusion, or vertical transmission from an infected pregnant woman to the fetus. After entry, during the primary stage of syphilis, the first manifestation involves the presentation of symptoms usually within 2 to 12 weeks after exposure to an infected person. This stage is characterized by the formation of a painless and hard lesion called a chancre, which normally appears on the genitals or mouth. In terms of nontreponemal tests, RPR and VDRL are two important clinical diagnostic methods; tests from the serum of patients with syphilis at different stages have low specificity and sensitivity [33]. The sensitivity of nontreponemal and treponemal tests for syphilis was increased with the duration of the disease, in the primary stage ranging from approximately 75%, and in the secondary stage to virtually 100% [34].

Currently, the detection of Treponema pallidum subspecies pallidum often involves serological testing to monitor disease progression, following CDC laboratory guidelines [35]. The traditional algorithm for syphilis serologic screening begins with a non-treponemal test (RPR/VDRL). Any reactive specimens are then confirmed with a treponemal test (TPHA/TPPA). This sequence has been widely used for decades due to the relatively low cost of non-treponemal tests. Alternatively, the reverse-sequence algorithm begins with an automated treponemal test, such as EIA or CIA. A positive result is followed by a quantitative non-treponemal test (RPR/VDRL). When the reverse-sequence algorithm yields discordant results, a second treponemal assay (TPHA/TPPA) is used for adjudication, involving a different format and antigen set. The reverse-sequence algorithm is becoming increasingly common in US laboratories. However, treponemal antibodies remain in the body for life after infection. This makes it difficult to distinguish between currently infected patients and patients who have previously been infected and treated. Both syphilis algorithms are used by laboratories. However, there are limited data on the performance and cost-effectiveness of the algorithms. The traditional algorithm may be less effective in detecting early or late latent syphilis, and using the reverse algorithm in low-prevalence populations might lead to an increase in false positives. Additionally, the CDC reported a false-positive rate for treponemal antibody tests (866 out of 140,176; 0.6%) when using the reverse-sequence algorithm from 2006 to 2010 [29]. Considering the limitations of *T. pallidum* detection methods, molecular assays using NAATs, such as PCR and LAMP assays, are employed for detection. NAATs amplifying the *Tpp47* gene by PCR-based methods showed high specificity (98–100%) and could be performed on different specimen types, including lesion exudates of primary and secondary syphilis; lesion biopsies of secondary syphilis; CSF from neurosyphilis cases; and whole blood, serum, and plasma from primary, secondary, and latent syphilis cases [29]. However, molecular biological techniques such as PCR have limitations as they require expensive equipment, long testing times, and lower sensitivity than LAMP. Therefore, the LAMP method requires only a water bath or heating block to obtain a constant temperature, which is an alternative molecular tool [21].

The LAMP-HNB technique developed in this study is a better method because the test results can be interpreted visually without the need for expensive equipment [36]. However, one of the major limitations of this assay compared to PCR is the complexity of designing LAMP primers. LAMP requires the design of at least two primer pairs to identify six regions of the target gene to increase the efficiency of the reaction. The HNB was found to be the best indicator as it is easy to prepare and inexpensive (approximately 0.0009 USD per 100 reactions) and provides reliable interpretation when compared with turbidity, the addition of calcein with MnCl_2_ (0.0011 USD) and Quant-iT Picogreen (DNA intercalating dye, 353.06 USD) [37]. LAMP-HNB can be optimized by adding HNB to the reaction solution at a final concentration of only 5 μM, less than that reported by Motoki Goto et al. The study on the colorimetric detection of LAMP using HNB with a final concentration of 120 μM solutions revealed a correlation with the Mg^2+^ ion concentration. The solutions including HNB in the absence of Mg2+ ions exhibited an absorbance peak at 650 nm, which lies within the wavelength range between 630 and 670 nm [23].

In this study, we developed a LAMP-HNB method for detecting T. pallidum subspp. pallidum. The LAMP method was custom-designed based on the gene sequence of the *Tpp47* gene. The LAMP-HNB technique offers convenience and can be completed in 60 min. When comparing the analytical sensitivity of LAMP-AGE and LAMP-HNB using 10-fold serial dilutions of DNA template from *T. pallidum* (ATCC BAA^®^2642SD^TM^), it was found that LAMP exhibited a 10-fold higher sensitivity than simple PCR. The analytical specificity of LAMP-AGE and LAMP-HNB showed no cross-reactivity with other bacteria, HIV, or the human genome in healthy human serum. We validated the LAMP assay for the diagnosis of syphilis using clinical samples. Both LAMP-AGE and LAMP-HNB assays demonstrated high sensitivity, with each reaching 100% sensitivity. The diagnostic specificity, PPV, and NPV of these assays were 91.00%, 91.74%, and 100.00%, respectively. Our study also showed that the sensitivity and NPV of LAMP-HNB were higher than in a previous report by Yongjian Xiao (2016–2017), who investigated a LAMP assay for the detection of T. pallidum DNA using the *bmp* gene [38,39]. Additionally, 18 clinical specimens tested positive with LAMP-HNB, while serological tests yielded negative results. We hypothesized that these patients might have been in the early stages of syphilis [40].

The syphilis algorithm may require additional confirmatory or response tests, meaning that laboratory serological testing for syphilis may take a long time and may require a return to the clinic for follow-up or treatment. An accurate point-of-care test (POCT) LAMP-HNB assay can reduce the time to diagnosis because it can identify infection during the first visit to the physician, allowing the patient to receive the appropriate treatment, which leads to reduced chances of progression to the late stages of the disease with severe symptoms and high mortality.

## 5. Conclusions

The LAMP-HNB assay is an important tool in managing syphilis patients, especially in areas with high prevalence but limited resources. It is a rapid, simple, highly sensitive, and highly specific test when used in conjunction with serological testing. Additionally, the LAMP-HNB assay does not require special equipment, such as a thermal cycler. In the future, the LAMP-HNB assay may develop into a single-step diagnostic process, enabling its use as point-of-care testing for the diagnosis, prevention, and management of syphilis infection.

## Figures and Tables

**Table 1:** The primers for the LAMP assay targeting the *Tpp47* gene.

Primer Name	Sequence (5′ -> 3′)
F3	5′-CAGGGCCGG GATACTACA-3′
B3	5′-TCG GCAAACACGTCAACT G-3′
FIP	5′-TTGGGAAGGAAGCGCACATCA-TTTTT-GGCTGACTTTGATTGCGA AC-3′
BIP	5′-ATTGGGTTGAAGGGGAAGGTGC-TTTTT-AGCATCCATCAGAGT CTCCG-3′

**Table 2:** The classification of the clinical serum samples.

Physician-Assessed Samples Classification	RPR Test	TPHA Test	Number of Clinical Serum Samples (*n*)
Disease	Reactive, ≥1:16	Reactive	200
Non-disease	Non-reactive	Non-reactive	200

**Table 3:** A 2 × 2 table for comparing results from a diagnostic test and true disease status. A 2 × 2 table with calculations: sensitivity = TP/(TP + FN), specificity = TN/(FP + TN), positive predictive value (PPV) = TP/(TP + FP), negative predictive value (NPV) = TN/(FN + TN).

		Disease Status	
		Disease	Non-Disease
Diagnostic Test	Positive	True positive (TP)	False positive (FP)
	Negative	False negative (FN)	True negative (TN)

**Table 4:** The classification of clinical serum samples.

Physician-Assessed Samples Classification	RPR Test	TPHA Test	Samples (*n*)	PCR-AGE	LAMP-AGE	LAMP-HNB
Disease	Reactive, ≥1:16	Reactive	200	Positive 200 (100%)	Positive 200 (100%)	Positive 200 (100%)
Non-disease	Non-reactive	N/A	200	Negative 189 (94.5%)	Negative 182 (91%)	Negative 182 (91%)

**Table 5:** A 2 × 2 table for comparing results from the PCR-AGE, LAMP-AGE, and LAMP-HNB tests and true disease status.

Method	Results	Disease Status	
		Disease	No Disease
PCR-AGE	Positive	200	11
	Negative	0	189
LAMP-AGE	Positive	200	18
	Negative	0	182
LAMP-HNB	Positive	200	18
	Negative	0	182

**Table 6:** The diagnostic accuracy analysis of PCR-AGE, LAMP-AGE, and LAMP-HNB (95% CI, *p* < 0.05).

Validity and Predictive Evaluation	PCR-AGE (95% CI)	LAMP-AGE (95% CI)	LAMP-HNB (95% CI)
Sensitivity	100.00% (100.00)%	100.00% (100.00)%	100.00% (100.00)%
Specificity	94.50% (94.40–94.60)%	91.00% (90.87–91.13)%	91.00% (90.87–91.13)%
PPV	94.79% (94.69–94.88)%	91.74% (91.63–91.86)%	91.74% (91.63–91.86)%
NPV	100.00% (100.00)%	100.00% (100.00)%	100.00% (100.00)%

## Data Availability

The data present in this study are available on request from the corresponding author.
